# Rhythmic Metabolism in Osteosarcopenia: Emerging Evidence, a Chronometabolomic Framework for the Bone–Muscle Unit, and Research Priorities

**DOI:** 10.3390/metabo16080547

**Published:** 2026-08-02

**Authors:** Yirui Chen, Hongxin Gui, Mingyuan Liu, Sitong Liu, Chang Liu, Yuqing Xue

**Affiliations:** 1 School of Public Health and Health Sciences, Tianjin University of Traditional Chinese Medicine, Tianjin 301617, China; ciandata@163.com (Y.C.); ghostcixi@163.com (H.G.); 15256982115@163.com (M.L.); sitong.liu@tjutcm.edu.cn (S.L.); 2 School of Sport Science, Beijing Sport University, Beijing 100084, China

**Keywords:** osteosarcopenia, chronometabolomics, rhythmic metabolites, circadian rhythm, bone–muscle crosstalk, time-targeted interventions

## Abstract

Osteosarcopenia combines low bone mass or skeletal fragility with sarcopenia, yet its metabolic biology is commonly assessed using single-time-point measurements. We conducted a structured narrative review of PubMed/MEDLINE, Web of Science Core Collection, Embase and Scopus from inception through 28 June 2026, supplemented by ScienceDirect, Google Scholar and citation chaining. Evidence was classified as direct human osteosarcopenia evidence, indirect human evidence, preclinical evidence or conceptual inference. Here, chronometabolomics denotes time-anchored or repeated metabolomic profiling used to estimate variation in phase, amplitude and pathway coordination in relation to sleep, feeding, activity and endocrine timing. Direct human evidence remains scarce. Component-specific human studies and experimental models suggest, but do not establish, that aging, chronic disease and irregular schedules may alter glucose–insulin, amino-acid, lipid, mitochondrial, redox-inflammatory, mineral-endocrine and microbial rhythms relevant to the bone–muscle unit. Chronometabolomics is therefore an emerging research framework, not a validated diagnostic or therapeutic approach. Daytime light, sleep regularization, protein distribution, time-restricted eating, exercise timing and medication timing remain candidates for controlled trials rather than current clinical recommendations; safety evaluation must address frailty, low body mass index, malnutrition, kidney disease, diabetes, polypharmacy, falls and inadequate protein or calcium intake.

## 1. Introduction

Osteosarcopenia describes the clinically important overlap between low bone mass or skeletal fragility and sarcopenia. It is associated with falls, fractures, disability, frailty and mortality, although current evidence does not establish that these risks exceed the additive effects of bone and muscle impairment considered separately [1]. No universally accepted case definition exists. Most studies operationalize osteosarcopenia as concurrent osteopenia or osteoporosis, usually assessed by dual-energy X-ray absorptiometry (DXA) or fragility-fracture criteria, and sarcopenia diagnosed using a named consensus algorithm, such as EWGSOP2 or AWGS 2019, that incorporates low muscle strength and low muscle quantity or quality, with poor physical performance used to indicate severity [2,3]. Definitions, thresholds and DXA sites vary across cohorts and should not be treated as interchangeable.

The bone–muscle unit provides a conceptual framework for studying the combined phenotype. Endocrine signals, inflammatory mediators, nutrient handling and tissue-derived secretory factors may interact across organs [4,5]. Endocrine crosstalk and RANKL-related signaling are plausible routes through which bone loss and muscle decline may reinforce one another [6,7], while broader reviews place the combined phenotype within metabolic aging, multimorbidity and functional decline [8,9].

Most explanatory models emphasize static abnormalities, including inflammation, mitochondrial dysfunction, altered sex-hormone exposure, glucocorticoid excess, insufficient protein intake, reduced mechanical loading and gut microbial dysbiosis. These mechanisms have empirical support to varying degrees, but their temporal organization is less well characterized. Glucocorticoid signaling illustrates the issue because local and systemic exposure may shape metabolism, muscle catabolism and bone remodeling in a phase-dependent manner [10,11]. In chronic kidney disease, cortisol dysregulation occurs alongside altered mineral metabolism and inflammation, making an untimed measurement difficult to interpret [12]. Bone turnover markers show diurnal variation [13], and the human metabolome changes across endogenous and behavioral cycles [14]. An unchanged daily mean may therefore coexist with altered amplitude, phase or exposure duration, although these features have not been validated as osteosarcopenia biomarkers.

The circadian timing system offers one organizing layer for this biology. Circadian timing has become an explicit topic in sarcopenia research [15], and sleep–circadian interactions are increasingly studied in relation to skeletal muscle metabolic health [16]. In healthy young men, experimental circadian misalignment altered the skeletal muscle lipidome [17]; 24-h profiling identified different muscle metabolic patterns in older, metabolically compromised men and young healthy men [18]; and age was associated with differences in the temporal structure of the plasma lipidome [19]. In observational studies of older adults, circulating metabolites were associated with rest–activity rhythm characteristics, but these findings do not establish that behavioral rhythm disruption caused the metabolic differences [20,21].

Exercise timing extends this concept from observation to intervention biology. A randomized crossover study in people with type 2 diabetes found time-of-day differences in multi-tissue metabolomic and skeletal muscle proteomic responses to exercise [22]. Human and experimental studies also associate resistance-exercise timing with BMAL1/PGC1α4–FNDC5/irisin signaling [23], diurnal variation in mitochondrial function and exercise capacity [24], and HIF1α-mediated substrate preference [25]. Complementary human profiling and preclinical studies implicate clock proteins in substrate metabolism and muscle performance [26,27,28,29,30]. These findings are not osteosarcopenia-specific and do not identify a universally beneficial exercise time.

Bone is also time-structured. Human evidence is most direct for diurnal variation in bone turnover markers and associations between rest–activity rhythms and osteoporosis-related outcomes [13,31]. Reviews and mechanistic studies connect clock biology with bone energy metabolism, endochondral formation, collagen hydroxylation and growth-plate biology [32,33,34,35]. Evidence involving endothelial BMAL1, muscle *Bmal1*, PER1 and glucocorticoid troughs is primarily preclinical [36,37,38,39]. Clock genes and microRNAs should therefore be viewed as research targets rather than established therapeutic targets in osteosarcopenia [40,41].

**Figure 1:**
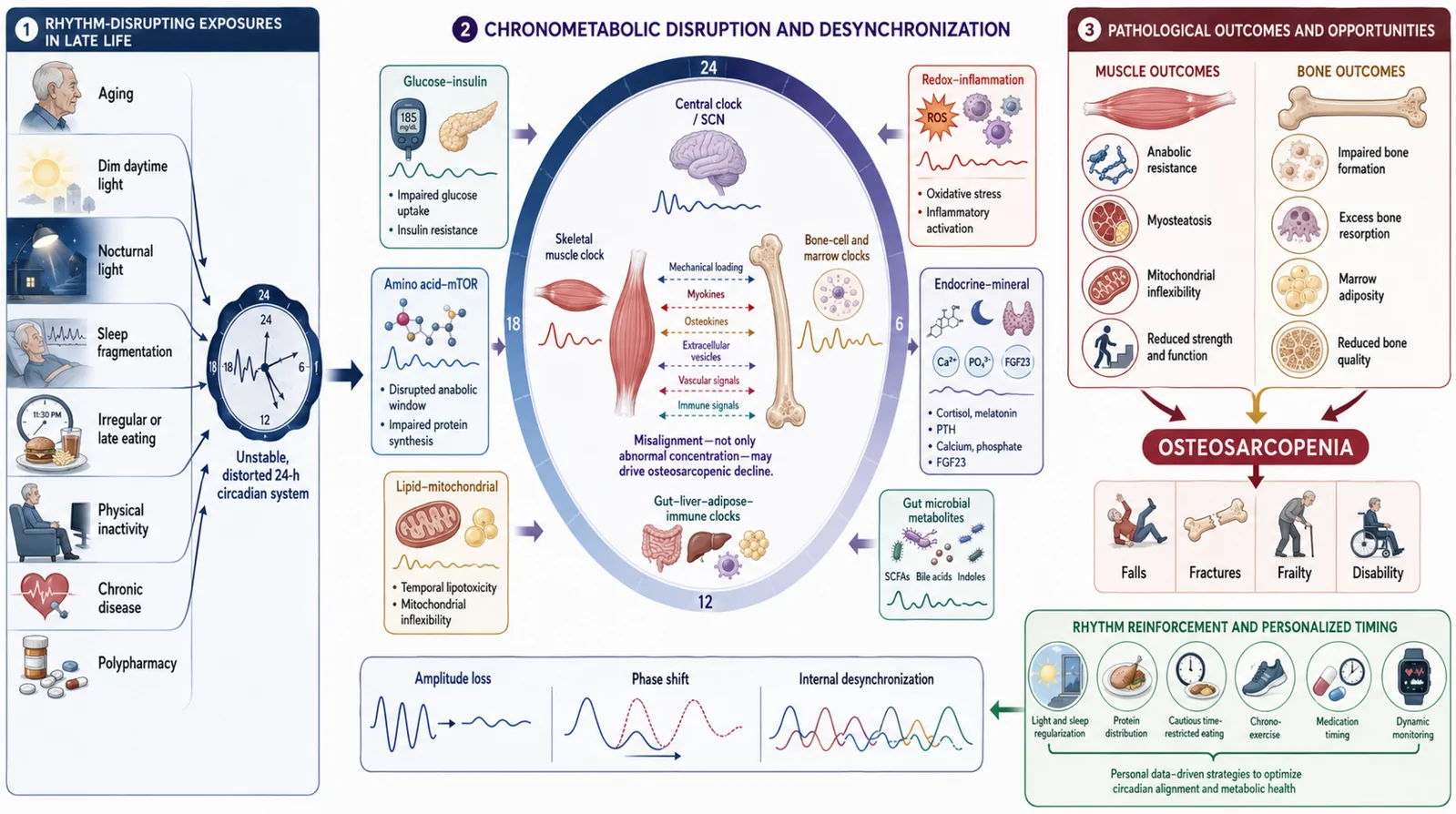
Hypothesis-generating framework for temporal metabolic disorganization of the bone–muscle unit. The arrows summarize proposed relationships among aging, chronic disease, behavioral schedules, metabolite timing and tissue communication; arrow direction indicates proposed influence or communication. Colors distinguish conceptual domains and do not encode evidence strength. The arrows do not demonstrate causality in osteosarcopenia. Evidence categories and limitations are summarized in Table 1.

We therefore examine osteosarcopenia through a hypothesis-generating framework of temporal metabolic disorganization within the bone–muscle unit (Figure 1). Here, chronometabolomics refers to time-anchored or repeated metabolomic profiling used to characterize variation in metabolite abundance and pathway behavior in relation to endogenous phase and behavioral cycles such as sleep, feeding and activity. We use circadian for endogenous approximately 24-h regulation, diurnal or time-of-day variation for patterns observed under ordinary environmental and behavioral conditions, and rest–activity rhythm for a behavioral proxy rather than a direct measure of endogenous phase. We evaluate whether aging, chronic disease and irregular behaviors may reshape the timing of amino-acid turnover, glucose utilization, lipid oxidation, mitochondrial energetics, redox balance, mineral metabolism and bone–muscle signaling. We do not treat this proposed temporal disorganization as an established mechanism of osteosarcopenia.

## 2. Review Design, Literature Search and Evidence Framework

This article is a structured narrative review rather than a systematic review or meta-analysis. Its conduct and reporting were informed by the Scale for the Assessment of Narrative Review Articles (SANRA) domains of justification, aims, search description, referencing, scientific reasoning and presentation of relevant data [42]. The review was not prospectively registered. PubMed/MEDLINE, Web of Science Core Collection (including Science Citation Index Expanded), Embase and Scopus were searched from database inception through 28 June 2026. ScienceDirect and Google Scholar were used as supplementary discovery platforms, and backward and forward citation chaining was used to locate relevant primary studies. Search concepts combined osteosarcopenia or bone–muscle phenotypes; circadian, sleep or behavioral timing; metabolomics or rhythmic metabolic pathways; tissue crosstalk; and time-targeted interventions. Appendix A reports source-specific search syntax and the role of each source.

Evidence was considered for inclusion when a peer-reviewed human observational or intervention study, repeated-sampling metabolomic or lipidomic study, or relevant experimental model addressed at least two of the following: a bone or muscle phenotype, endogenous or behavioral timing, metabolic or tissue-crosstalk pathways, and a time-targeted intervention. Reviews and consensus statements were used for context and citation discovery, with primary studies preferred for evidentiary claims. Duplicate reports, publications without a relevant bone or muscle outcome, reports without a temporal or metabolic component, and conference abstracts, editorials or isolated case reports were not used as primary evidence. No publication-date restriction was imposed; recent work from 2021–2026 was prioritized, while earlier studies were retained when required for foundational concepts.

Titles and abstracts were assessed for relevance, followed by full-text assessment of potentially informative reports. Selection was purposive and prioritized directness to osteosarcopenia, human relevance, study design, temporal resolution, methodological detail and the ability to address a defined mechanistic or translational question. The search did not produce an auditable, deduplicated record log suitable for a PRISMA flow diagram. Duplicate independent screening, formal risk-of-bias assessment and quantitative certainty grading were not performed; these features distinguish this narrative synthesis from a systematic review and are acknowledged as limitations. We did not reconstruct record counts retrospectively because doing so would imply a level of systematic-review reproducibility that the original search was not designed to provide.

Evidence was interpreted using four categories. Tier 1 denotes direct human evidence in participants with a defined osteosarcopenia phenotype and a relevant temporal measure. Tier 2 denotes indirect human evidence from sarcopenia, osteoporosis, frailty, sleep, metabolic or other informative cohorts. Tier 3 denotes preclinical or mechanistic evidence from animal, tissue or cellular models. Tier 4 denotes conceptual inference that has not been tested directly. Throughout the review, “associated with” is used for observational findings, “supports” is reserved for reproducible evidence within the studied population and design, and “may” or “hypothesized” identifies extrapolation. We identified no human study that simultaneously assessed a defined osteosarcopenia phenotype, a validated endogenous circadian phase marker and serial metabolomic profiles.

The literature is broad but uneven. Evidence is relatively more extensive for muscle metabolic timing, bone turnover-marker variation, rest–activity associations, nutrient timing and exercise timing than for their joint study in osteosarcopenia. This description reflects breadth and directness, not a formal certainty rating. Table 1 summarizes the evidence categories, osteosarcopenia-specific status and main interpretive boundaries.

**Table 1:** Evidence map for a chronometabolomic research framework in osteosarcopenia.

Domain	Representative Evidence	Tier	Status in Osteosarcopenia	Main Boundary
Osteosarcopenia phenotype	Burden and crosstalk syntheses [1,4]; endocrine and RANKL pathways [6,7]	1–3	Combined phenotype established; temporal model unvalidated	No serial chronometabolomic cohort
Muscle metabolic timing	Muscle lipidome and 24-h metabolome studies [17,18]; aging lipidome [19]	2–3	Candidate mechanisms	Mainly non-osteosarcopenia cohorts
Bone timing biology	Bone-marker variation [13]; bone clock reviews and models [32,33,36,38]	2–3	Candidate mechanisms	Human causal timing trials limited
Behavioral rhythms	Rest–activity metabolomics [20,21]; sleep, frailty and osteoporosis cohorts [43,44,45]	2	Candidate correlates	Confounding; reverse causality; phase not directly measured
Timed interventions	Exercise timing [22,23]; nutrient timing and time-restricted eating (TRE) [46,47]; bone-focused studies [48,49]	2–3	Investigational; not clinically validated	Safety and phenotype dependence
Microbial, redox and endocrine pathways	Melatonin and bone [50,51]; microbial timing [52,53]; redox and mitochondria [54,55]	2–4	Mostly speculative	Predominantly indirect or preclinical

Tier 1, direct human osteosarcopenia evidence; Tier 2, indirect human evidence; Tier 3, preclinical or mechanistic evidence; Tier 4, conceptual inference. “Unvalidated” means not validated for osteosarcopenia diagnosis, prognosis or treatment selection.

## 3. Temporal Organization of the Bone–Muscle Unit

The suprachiasmatic nucleus coordinates physiology with the light–dark cycle, while peripheral tissues contain local clocks influenced by feeding, physical activity, endocrine exposure, body temperature and autonomic inputs. Skeletal muscle, bone, liver, intestine, kidney, immune cells and vascular endothelium can therefore exhibit tissue-specific temporal patterns. External cues may transduce timing information to peripheral molecular clocks [56]. Whether loss of coordination among these systems contributes to osteosarcopenia remains an open question.

Skeletal muscle is a major site of glucose disposal, amino-acid turnover and fatty-acid oxidation. Experimental studies implicate the skeletal-muscle clock in insulin sensitivity, substrate handling, mitochondrial function, protein turnover and myokine secretion, although evidence for several downstream processes remains preclinical. Experimental circadian misalignment altered the muscle lipidome in healthy young men [17], while 24-h profiling identified different metabolic patterns in older, metabolically compromised men and young controls [18]. Exercise timing also modified metabolomic and skeletal muscle proteomic responses in type 2 diabetes [22]. These studies support standardizing sampling time but do not establish circadian causality in osteosarcopenia.

Bone also has substantial temporal organization. Bone turnover markers show diurnal variation in humans [13], and experimental studies connect clock biology with endochondral bone formation, collagen hydroxylation, endothelial support and inflammatory osteoclastogenesis [34,35,36,37]. A daily trough in glucocorticoid signaling appears important for skeletal health in mice [39]. Prospective human evidence associates rest–activity rhythms with osteoporosis risk [31]. These observations motivate testing whether altered remodeling timing contributes to bone loss; they do not show that such disruption occurs in osteosarcopenia.

**Figure 2:**
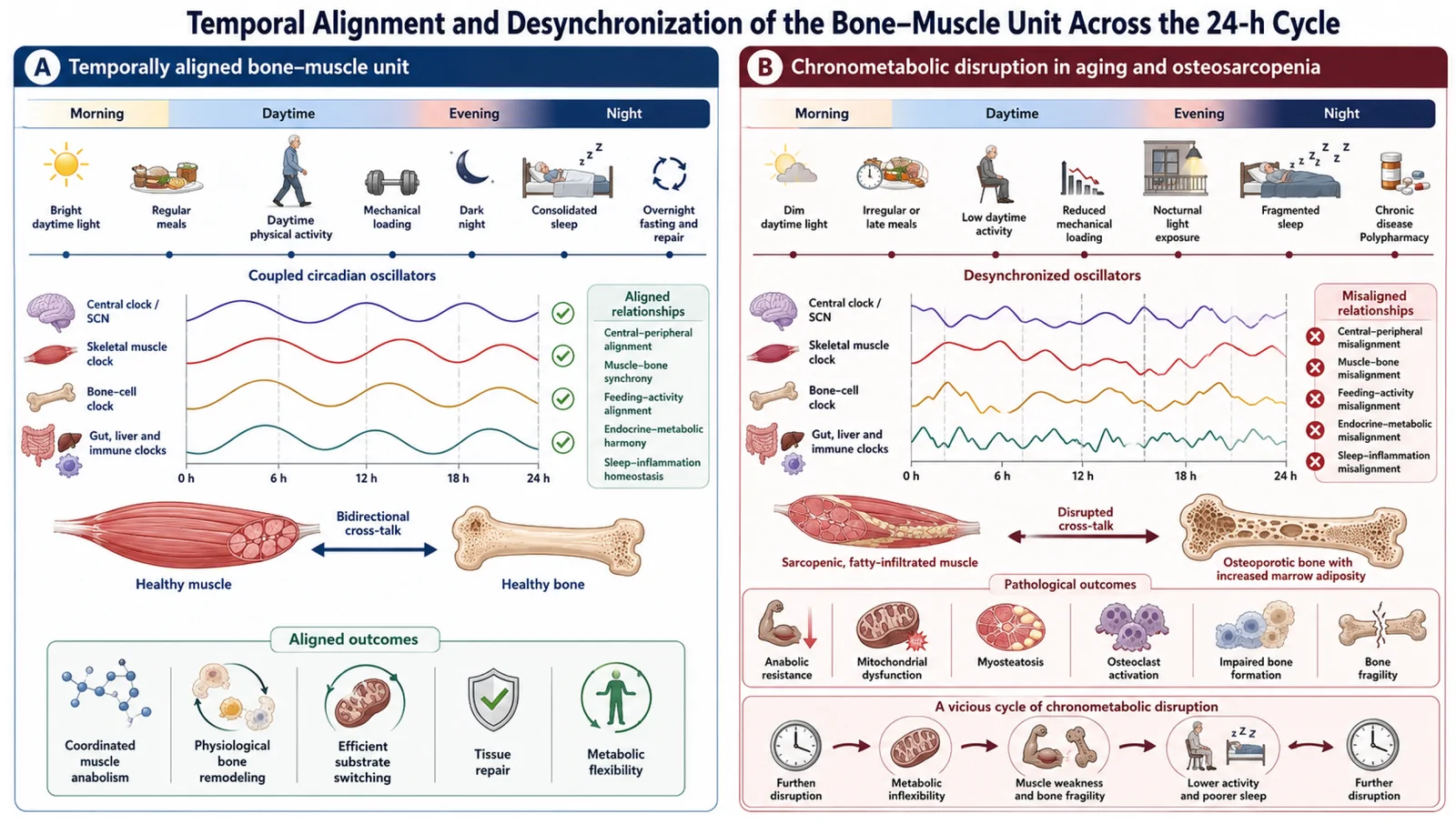
Conceptual comparison of temporal alignment and misalignment across the 24-h day. The schematic contrasts coordinated light, sleep, feeding and activity schedules with proposed endocrine and metabolic consequences of irregular schedules. Lines represent schematic temporal patterns; colors distinguish tissue or domain traces and do not encode measured effect sizes. It does not establish endogenous circadian misalignment or causality in osteosarcopenia.

Aging and chronic disease are associated with reduced rhythm amplitude, fragmented sleep–wake cycles, lower daytime light, nocturnal light exposure, altered melatonin and cortisol profiles, irregular meals, reduced activity amplitude and polypharmacy. Under the proposed model, these factors may produce misalignment between environmental, behavioral and tissue-level timing. Late eating may shift metabolic responses relative to activity; reduced outdoor activity may weaken both light exposure and mechanical loading; and sleep fragmentation may alter endocrine or inflammatory exposure. These examples are hypotheses that require direct testing in osteosarcopenia (Figure 2).

## 4. Chronometabolic Pathways in Osteosarcopenia

**Figure 3:**
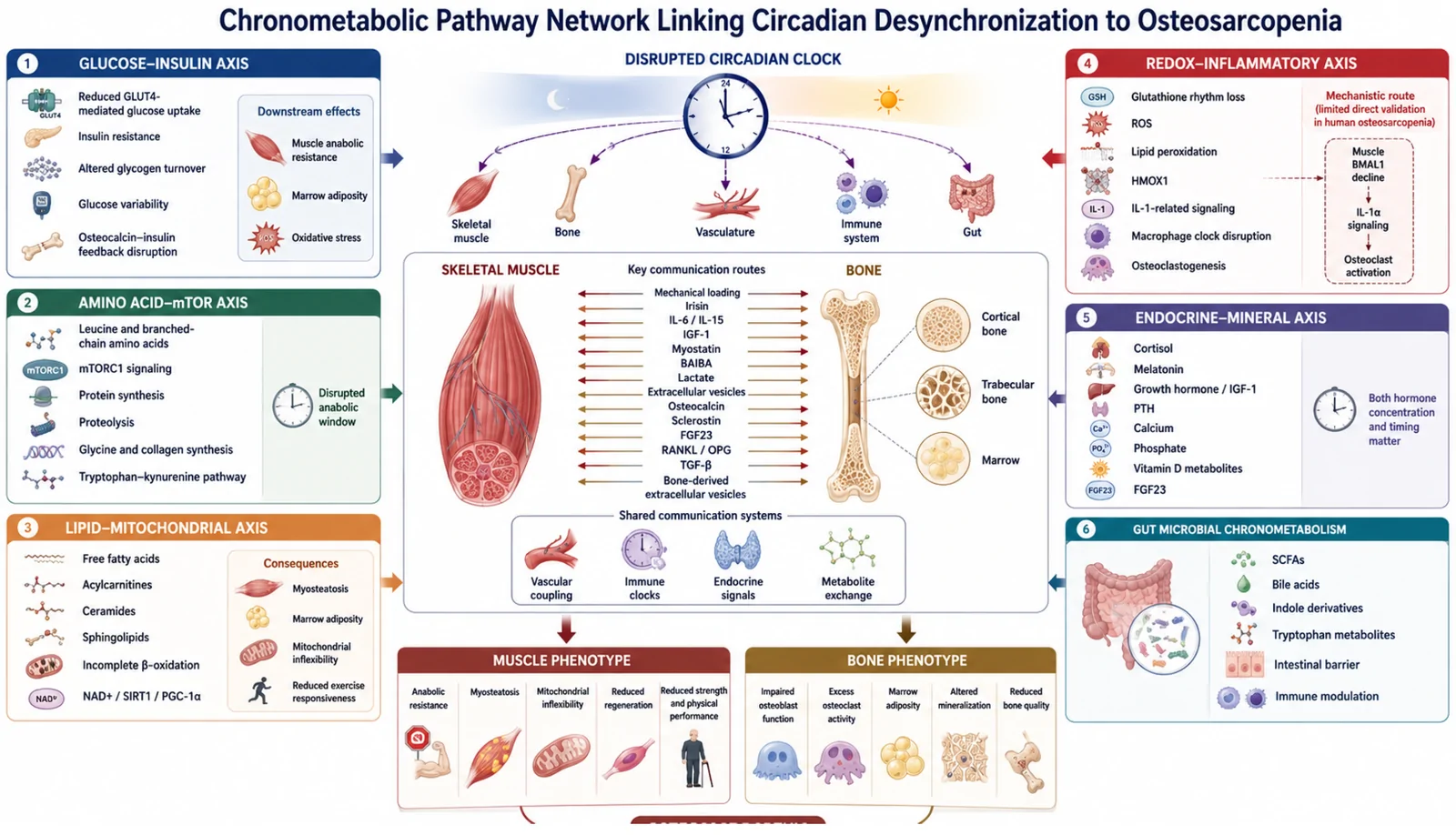
Hypothesis-generating chronometabolic pathway network. The schematic integrates glucose–insulin handling, amino-acid availability, lipid remodeling, mitochondrial energetics, redox-inflammatory signaling, endocrine–mineral timing and microbial metabolites. Solid arrows indicate proposed direction of influence or bone–muscle communication, whereas dashed arrows indicate indirect or putative links; colors distinguish pathway axes and do not encode evidence strength. Most links are supported by indirect human or preclinical evidence and should not be interpreted as established osteosarcopenia mechanisms.

A temporal perspective recognizes that disease-relevant metabolites can vary with endogenous phase, clock time and behavior. A pathway may differ in its mean level, within-day range, estimated phase or relationship to feeding and activity, but those features require appropriate sampling and cannot be inferred from a single measurement. Figure 3 summarizes the proposed network, and Table 2 identifies candidate readouts, evidence tiers and osteosarcopenia-specific validation status.

Glucose and insulin provide an informative example because skeletal muscle accounts for substantial postprandial glucose disposal, while bone participates in energy metabolism through osteocalcin, marrow adiposity and osteoblast energy demand. Human studies demonstrate morning-to-evening differences in metabolic state [63], whereas causal evidence for tissue-clock control of glucose tolerance comes mainly from mouse models [57,58]. Whether meal, sleep or activity timing produces a distinct glucose-related phenotype in osteosarcopenia remains unknown; these variables should be recorded as potential modifiers.

Amino-acid and protein timing may link nutrition to both tissues. Muscle protein synthesis depends on amino-acid availability, mTORC1 signaling, insulin permissiveness and recent contractile activity, whereas bone matrix formation requires collagen substrates and an appropriate endocrine and energetic context. Branched-chain amino-acid catabolism is central to skeletal muscle and whole-body metabolism [59]. Total daily protein intake does not indicate how protein is distributed across meals or aligned with exercise, and whether such distribution alters bone–muscle outcomes in osteosarcopenia has not been directly tested.

Lipid and mitochondrial patterns provide a second bridge between metabolic disease and the proposed framework. Experimental circadian misalignment altered skeletal muscle lipid composition in healthy young men [17], and age was associated with differences in the temporal plasma lipidome [19]. Evidence that muscle clocks influence mitochondrial dynamics is largely mechanistic [60], and inactive-phase time-restricted feeding abolished daily variation in mitochondrial respiration in rat skeletal muscle [55]. A single fasting sample cannot characterize diurnal variation, but the clinical value of repeated lipid or mitochondrial measurements in osteosarcopenia remains unestablished.

Redox, mineral-endocrine and microbial pathways extend the range of candidate mechanisms. Redox homeostasis is associated with skeletal muscle aging [54], while glucocorticoid timing may influence muscle insulin sensitivity and skeletal remodeling [11,64]. Melatonin has been studied in relation to osteoblastogenesis, osteoclastogenesis and oxidative stress [50,65], and urinary 6-sulfatoxymelatonin rhythms were associated with a bone-resorption marker in blind women [51]. Gut microbial metabolites may transmit feeding-time signals to immune and bone pathways [52,53]. A recent cardiometabolic narrative review links gut-derived metabolites with mitochondrial redox dysfunction and ferroptosis but treats the complete pathway as hypothesis-generating; it therefore informs mechanism selection rather than osteosarcopenia-specific inference [66]. Most evidence remains indirect or preclinical for osteosarcopenia; these axes are candidate or speculative mechanisms, not clinical biomarkers.

## 5. Temporal Bone–Muscle Crosstalk

The bone–muscle unit communicates through mechanical load, endocrine and paracrine factors, metabolites, extracellular vesicles, neural inputs, vascular supply and immune mediators. A temporal perspective raises four questions: when a signal is produced, when it is released, whether receptor sensitivity varies over time and whether target-tissue responses depend on biological or behavioral context. Table 3 summarizes the proposed routes and their evidentiary limits.

Mechanical loading is the most familiar bone–muscle signal. Exercise produces strain, myokine release, lactate flux, vascular shear, amino-acid demand and changes in insulin sensitivity. Bone responses to loading may differ with nutritional, endocrine and sleep context; time-of-day-dependent skeletal effects have so far been demonstrated mainly in mice [49]. Direct clinical testing is required before extrapolating an optimal loading time to osteosarcopenia.

Secretory crosstalk may also be time-dependent. Myokines such as irisin, IL-6, IL-15, myostatin and IGF-1, and osteokines such as osteocalcin, sclerostin and FGF23, are influenced by activity, feeding, fasting, sleep and inflammation. The BMAL1/PGC1α4–FNDC5/irisin axis has been implicated in time-of-day resistance-exercise responses [23], while RANKL-related signaling is a plausible link between bone and muscle decline [7]. Most human studies use one sampling time and cannot distinguish reduced production from altered timing.

Immune, vascular, endocrine and microbial routes broaden the model. Endothelial BMAL1 decline contributed to age-related bone loss in mice [36]; aging-related muscle *Bmal1* decline enhanced IL-1α-mediated osteoclastogenesis in a mouse model [37]; and gut microbial metabolites have been implicated in experimental temporal regulation of bone homeostasis [52]. These findings generate mechanistic hypotheses but do not identify validated biomarkers or therapeutic targets in people with osteosarcopenia.

## 6. Chronometabolomics: Candidate Biomarkers and Study Designs

**Figure 4:**
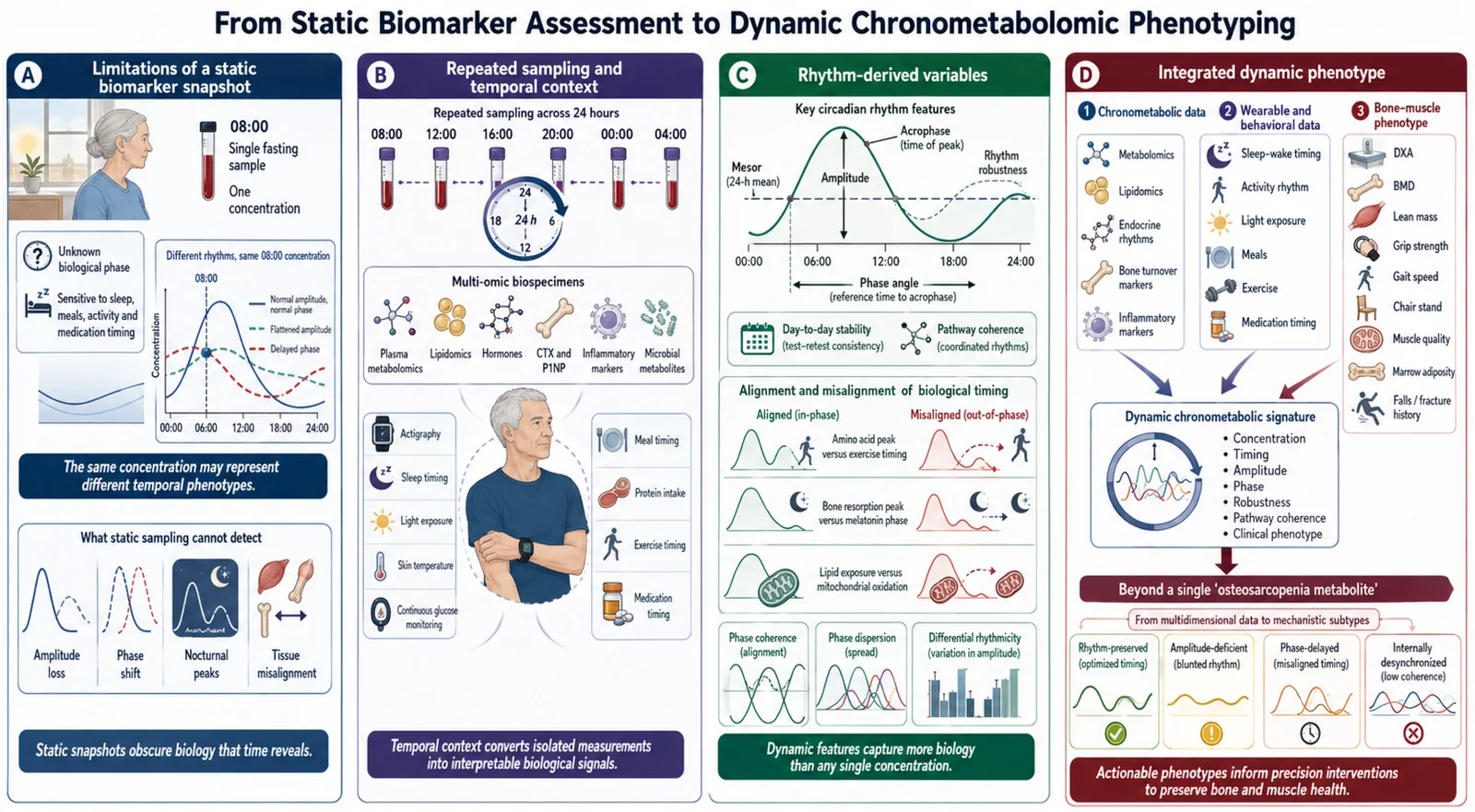
Research design for moving from a single measurement to time-anchored metabolic profiling. Arrows show the proposed information flow from temporal context and repeated sampling to an integrated phenotype; colored traces illustrate example rhythmic profiles and do not represent measured patient data. Repeated sampling can be used to estimate within-day range, timing, robustness, area under the curve and pathway coherence. Sparse or behaviorally uncontrolled sampling does not by itself establish endogenous circadian phase or tissue alignment.

A standardized single measurement of glucose, C-terminal telopeptide of type I collagen (CTX), procollagen type I N-terminal propeptide (P1NP), cortisol, amino acids or inflammatory markers may be clinically useful, but it cannot characterize within-day variation. Chronometabolomic research combines time-anchored or repeated metabolite sampling with information on sleep, light, meals, activity and medication. Repeated circulating measurements may estimate diurnal range, timing and robustness; demonstrating alignment between tissues requires tissue-specific measurements or validated proxies, and demonstrating endogenous circadian phase requires an appropriate marker or controlled protocol. Figure 4 and Table 4 summarize these distinctions.

A pragmatic design does not always require around-the-clock sampling. At minimum, investigators should standardize sampling time and report fasting duration, prior meal timing, sleep timing, recent activity and medication timing. Multiple daytime samples can characterize within-day variation, whereas dense 24-h sampling or a validated phase marker is required for robust circadian phase or amplitude estimation. Rest–activity studies in older women and men show that metabolites can track behavioral rhythm features [20,21]; actigraphy can therefore contextualize metabolomics but should not be treated as a direct measure of endogenous phase.

Sleep duration, sleep quality and sleep disorders have been associated with sarcopenia or muscle loss [43,67,68]; sleep quality, objective sleep parameters and sleep regularity have been associated with osteoporosis-related outcomes [44,69,70]; and cortisol rhythm has been associated with sarcopenia in patients with type 2 diabetes [71]. These findings are vulnerable to confounding, reverse causality and inconsistent timing metadata. They do not establish that sleep disruption causes osteosarcopenia or that rhythmic metabolites mediate the observed relationships. Complementary pathway-level blood metabolomics in cognitive aging associated lifestyle-related energy, amino-acid, lipid, inflammatory/redox and microbiome-linked patterns with grip strength, gait speed and frailty [72]. This supports integrating functional outcomes into metabolomic studies, but it neither evaluates temporal profiles nor validates biomarkers in osteosarcopenia. Future studies should pair clinical phenotyping with repeated metabolite sampling, objective sleep/activity data and medication and nutrition records.

## 7. Candidate Time-Targeted Interventions: Research Rationale and Safety

None of the timing strategies discussed in this section has been validated as an osteosarcopenia-specific treatment. Their inclusion does not constitute clinical advice or support a universal optimal treatment time. Older adults differ by chronotype, frailty, medication schedule, comorbidities, appetite, fall risk, caregiving environment and social constraints. Light, diet, exercise and medication timing can be evaluated as coordinated research exposures [73], but established osteoporosis and sarcopenia care should not be delayed or replaced. Table 5 summarizes the evidence status, clinical readiness and principal safeguards.

Chrononutrition requires particular caution. Energy-intake timing is being discussed as a candidate modifier of aging biology [74], and a short-term crossover trial in adults with type 2 diabetes reported improved glucose homeostasis, but not improved insulin sensitivity, during time-restricted eating [47]. Bone-focused evidence remains mixed, and changes accompanying weight loss complicate interpretation [48,75]. Any osteosarcopenia trial should prespecify safeguards against inadequate energy, protein, calcium and micronutrient intake and should exclude or closely supervise participants with frailty, low body mass index, malnutrition risk, kidney disease or glucose-lowering medication. Protein distribution, meal quality and coordination with exercise are higher-priority trial questions than fasting duration alone.

Exercise timing is also investigational. Human evidence comes largely from type 2 diabetes or other populations without defined osteosarcopenia, while several mechanistic findings derive from experimental models [22,23,24,25,76,77]. Reviews discuss exercise as a potential timing cue for peripheral clocks [78,79], but the safest feasible time should be individualized around chronotype, cardiovascular status, fatigue, sleep and fall risk. Exercise itself remains central to muscle, balance and bone health; current data justify osteosarcopenia-specific timing trials, not a single recommended exercise hour.

Medication schedules should not be changed by patients on the basis of this framework. Timing studies must be indication-specific and clinician-led, account for kidney function, diabetes treatment, glucocorticoid exposure and polypharmacy, and distinguish pharmacokinetic convenience from improved bone–muscle outcomes.

**Figure 5:**
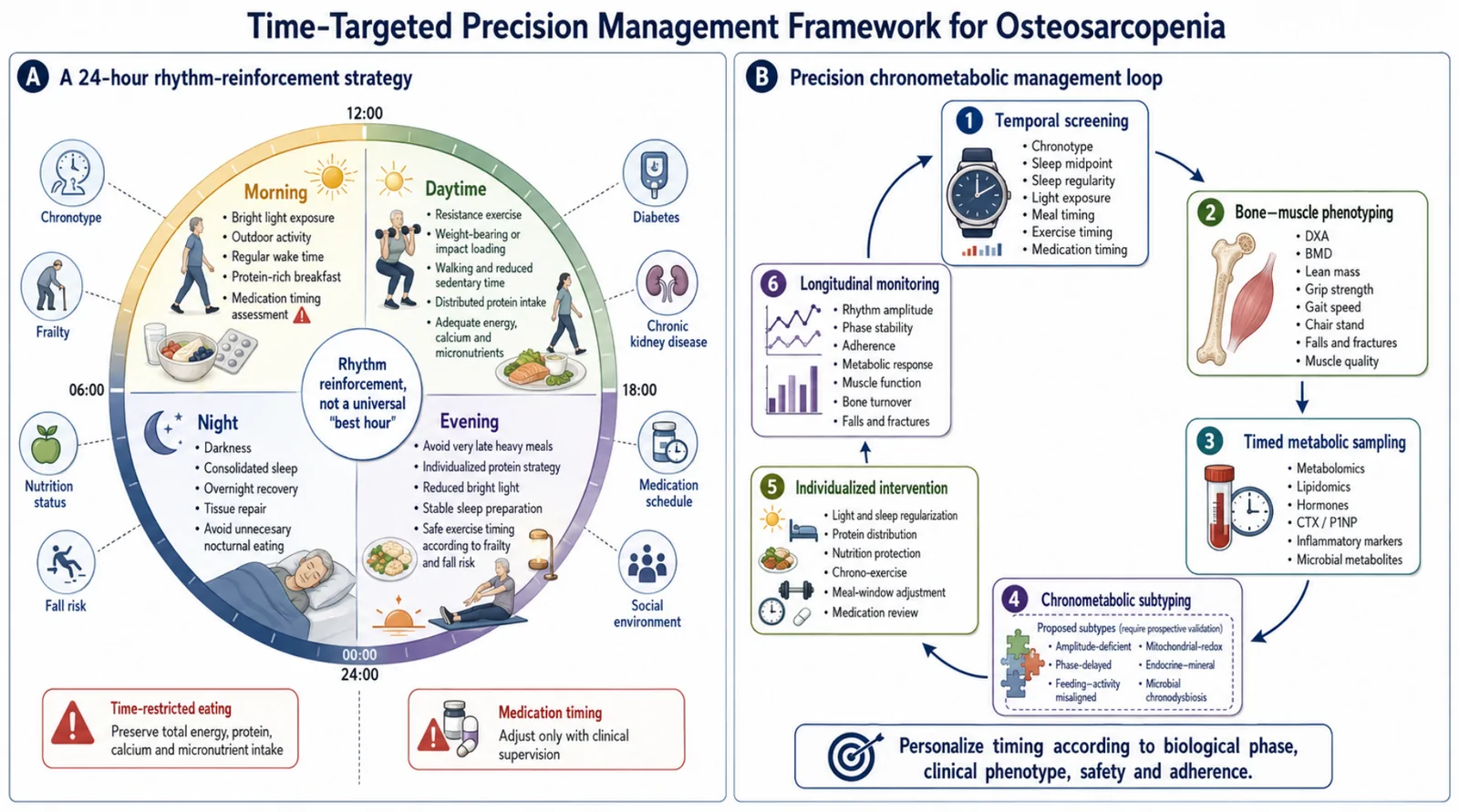
Hypothesis-generating research framework for evaluating time-targeted strategies. Arrows indicate the proposed evaluation loop; colors separate workflow stages and do not encode evidence strength. Temporal characterization, standard bone–muscle phenotyping, time-anchored metabolic assessment and longitudinal monitoring may be linked in future trials. This is not a validated management pathway, diagnostic algorithm or recommendation to alter treatment timing.

Figure 5 summarizes this proposed, investigational workflow.

## 8. Discussion

### 8.1. Interpretation of the Evidence

The principal finding of this review is a mismatch between biological plausibility and disease-specific evidence. Human studies demonstrate time-of-day variation in bone turnover markers and skeletal muscle metabolism and show associations between behavioral rhythms and bone or muscle outcomes. Experimental models identify plausible clock-dependent pathways. However, no identified human study combined a defined osteosarcopenia phenotype, a validated endogenous phase marker and serial metabolomic profiling. The proposed framework is therefore integrative and hypothesis-generating; it does not establish a distinct causal subtype of osteosarcopenia.

Several uncertainties limit synthesis. Circadian, diurnal and rest–activity measures are not interchangeable. Metabolite timing depends on chronotype, light, meals, acute exercise, posture, sleep, medication and comorbidity, while cross-sectional associations cannot distinguish cause from consequence. Evidence on time-restricted eating and skeletal outcomes is mixed and may be confounded by weight loss or reduced nutrient intake. Exercise studies differ in population, mode, intensity and timing reference, and they do not support a universal optimal time. Tissue-specific clock-gene models establish mechanistic possibilities but may not reproduce multimorbidity, frailty or polypharmacy in older adults.

### 8.2. Incremental Clinical Value and Feasibility

Chronometabolomics must demonstrate value beyond existing assessment. DXA, fracture history and risk tools, grip strength, chair-rise and gait-speed tests, lean-mass measures, routine laboratory tests and standardized bone-turnover markers already support diagnosis and risk stratification. Rhythm-derived variables would be clinically useful only if they improve diagnosis, prognosis, treatment selection or longitudinal monitoring beyond these tools with acceptable cost and participant burden. At present, chronometabolomics should neither replace established diagnostic criteria nor delay evidence-based osteoporosis, nutrition, exercise or fall-prevention care.

Repeated sampling introduces participant burden, possible sleep disturbance, incomplete adherence, assay batch effects, cost and unstable phase estimates from sparse observations. A staged strategy may be more feasible: standardized single-time sampling for routine comparability; several targeted daytime samples for within-day variation; and dense 24-h sampling or validated phase markers for mechanistic circadian studies. Home or minimally invasive sampling may reduce burden but requires analytical validation. Protocols should prespecify temporal endpoints and distinguish clock time, biological phase and behavior.

### 8.3. Research Priorities

Direct cohorts should include healthy controls, isolated osteoporosis or osteopenia, isolated sarcopenia and combined osteosarcopenia. Participants should be characterized by sex, menopausal status, diabetes, kidney function, glucocorticoid exposure, medications, chronotype, sleep disorders, physical activity and nutrition. Timing metadata should be treated as primary data, and candidate signatures should be externally validated against clinically meaningful outcomes.

Triangulation across tissue-specific models, longitudinal cohorts, randomized trials and Mendelian-randomization analyses may strengthen causal inference, although each design has distinct assumptions and limitations. Trials should test whether time-targeted strategies add benefit beyond adequate protein and calcium intake, resistance and balance exercise, fall prevention and standard pharmacological care. Feasibility, safety, adherence, quality of life, falls and fractures should accompany metabolomic and rhythm endpoints.

## 9. Limitations of This Review

This review has several limitations. It is a structured narrative review rather than a systematic review; no prospective protocol, exhaustive record-level screening log, quantitative synthesis, formal risk-of-bias assessment or certainty-of-evidence evaluation was undertaken. Purposive selection of representative studies may have introduced selection and citation bias. Definitions of osteosarcopenia, sarcopenia and osteoporosis, as well as populations, sampling schedules, analytical platforms and outcomes, were heterogeneous. Much of the evidence was extrapolated from component disorders, metabolically compromised populations or experimental models, and most proposed biomarkers and interventions lack osteosarcopenia-specific validation. The search emphasized English-language bibliographic interfaces and may not have captured all regional or non-indexed literature. The rapidly evolving literature may also alter the balance of evidence. These limitations preclude causal inference, definition of diagnostic thresholds or recommendation of optimal intervention times.

## 10. Conclusions

Chronometabolomics is a promising research framework for testing whether temporal metabolic disorganization contributes to coupled bone and muscle decline. Current evidence is dominated by component-specific human studies and mechanistic or preclinical models; direct osteosarcopenia studies with repeated temporal profiling remain insufficient to establish diagnostic biomarkers, causal pathways or optimal treatment timing. Future studies should combine consensus bone–muscle phenotyping, standardized timing metadata, repeated or targeted sampling and safety-focused prospective trials, and should test whether rhythm-derived measures add value beyond existing clinical tools. Until such evidence is available, time-targeted interventions should remain individualized research candidates rather than routine recommendations for osteosarcopenia.

## Figures and Tables

**Table 2:** Candidate chronometabolic pathways and readouts relevant to osteosarcopenia.

Axis	Candidate Time-Sensitive Readouts	Putative Bone–Muscle Relevance	Tier	Osteosarcopenia Status
Glucose–insulin	Glucose, insulin, lactate, pyruvate, continuous glucose monitoring variability	Energy supply, marrow adiposity, glucose disposal and anabolic permissiveness [57,58]	2–3	Candidate; unvalidated
Amino acids	Leucine, branched-chain amino acids (BCAAs), glycine, arginine, tryptophan, 3-methylhistidine	Collagen substrates, protein synthesis and proteolysis [46,59]	2–3	Candidate; unvalidated
Lipids	Free fatty acids, acylcarnitines, phosphatidylcholines, ceramides	Marrow adiposity, myosteatosis, oxidation and insulin sensitivity [17,19]	2	Candidate; unvalidated
Mitochondria	NAD^+^/NADH, citrate, succinate, beta-hydroxybutyrate, ATP-linked flux	Cell differentiation, oxidative capacity, exercise tolerance and repair [60,61]	2–3	Candidate; largely preclinical
Redox–inflammation	Glutathione, cysteine, lipid-peroxidation products, IL-1, IL-6 and TNF-α	Osteoclastogenesis, proteostasis and mitochondrial stress [37,54]	3	Speculative
Mineral–endocrine	Calcium, phosphate, parathyroid hormone, cortisol, melatonin, vitamin D metabolites and FGF23	Remodeling, mineralization and neuromuscular function [13,51]	2–3	Candidate; unvalidated
Microbial metabolites	Short-chain fatty acids, bile acids, indoles and trimethylamine-related metabolites	Immune tone, calcium handling and substrate metabolism [52,62]	3–4	Speculative

No readout in this table is validated for osteosarcopenia diagnosis, prognosis or timing-based treatment selection. Tier definitions are provided in Table 1.

**Table 3:** Hypothesis-generating temporal bone–muscle crosstalk routes and candidate readouts.

Route	Timing-Sensitive Feature	Candidate Readouts	Tier/Status	Main Limitation
Mechanical loading	Activity timing, loading frequency and recovery sleep	Activity timing, impact counts, lactate, CTX, P1NP and sclerostin	Tier 2–3; candidate	Optimal timing unknown
Myokines	Exercise-related release of irisin, IL-6, IL-15, IGF-1, myostatin and BAIBA	Timed myokines, metabolomics, grip strength, gait speed and DXA	Tier 2–3; candidate	Assay and context variability
Osteokines	Time-dependent osteocalcin, sclerostin, RANKL/OPG and FGF23 signaling	CTX, P1NP, osteocalcin, sclerostin, FGF23 and phosphate	Tier 2–3; candidate	Human muscle effects unclear
Vascular coupling	Endothelial timing and perfusion	Blood-pressure rhythm, pulse-wave velocity, muscle oxygenation and imaging	Tier 2–3; speculative	Activity confounding
Immune/redox coupling	Cytokine, immune-cell and antioxidant timing	IL-1, IL-6, TNF-α, C-reactive protein, glutathione and lipid peroxidation	Tier 3; speculative	Dense human sampling rare
Extracellular vesicles	Release and uptake after exercise, fasting or inflammation	Vesicle concentration, cargo profiling and paired metabolomics	Tier 3–4; speculative	Temporal human data scarce
Gut-derived signals	Feeding window, stool timing and microbial oscillation	Stool/plasma metabolomics, microbiome sequencing and meal logs	Tier 3–4; speculative	Timing metadata essential

BAIBA, beta-aminoisobutyric acid; CTX, C-terminal telopeptide of type I collagen; DXA, dual-energy X-ray absorptiometry; FGF23, fibroblast growth factor 23; IGF-1, insulin-like growth factor 1; OPG, osteoprotegerin; P1NP, procollagen type I N-terminal propeptide; RANKL, receptor activator of nuclear factor-kappa B ligand.

**Table 4:** Design priorities for chronometabolomic studies of osteosarcopenia.

Component	Minimum Reporting	Preferred Approach	Reason
Phenotype	Named osteoporosis/osteopenia and sarcopenia criteria; DXA site; strength and performance tests	Controls plus isolated bone loss, isolated sarcopenia and combined osteosarcopenia	Separates shared from component-specific patterns
Sampling time	Clock time, fasting duration, prior meal, posture, medication and recent activity	Multiple daytime samples for within-day variation; dense 24-h sampling for rhythm estimation	Avoids claiming phase or amplitude from sparse data
Biological phase	Distinguish clock time from biological time	Dim-light melatonin onset or another validated phase marker when circadian inference is intended	Tests endogenous phase rather than behavioral timing alone
Behavioral context	Sleep midpoint, light exposure, meal and exercise timing	Actigraphy, light sensor, meal logs and continuous glucose monitoring	Contextualizes, but does not directly measure, circadian phase
Assays	Targeted metabolomics, lipidomics, bone turnover and endocrine markers	Integrated metabolomics with proteomics, microbiome or transcriptomics	Links pathways to candidate communication routes
Analysis	Prespecified variables, covariates, missing data and multiple-testing control	Validated rhythm models, sensitivity analyses and longitudinal designs	Reduces false timing claims
Clinical utility	DXA, fracture risk, grip strength, gait speed and routine markers	Test incremental discrimination, prediction and responsiveness	Establishes value beyond existing tools
Safety/feasibility	Falls, appetite, weight, sleep disruption, burden and adverse events	Embedded adherence, cost and acceptability assessment	Essential in older or frail adults

**Table 5:** Hypothesis-generating time-targeted strategies for future osteosarcopenia trials; these are not current clinical recommendations.

Strategy	Research Premise	Evidence/Status	Clinical Readiness	Principal Safeguards
Daytime light	Brighter days and darker nights may reinforce sleep–activity timing	Tier 2–3; candidate	Osteosarcopenia benefit unvalidated	Eye disease, photosensitive drugs and inappropriate phase shifting
Sleep regularity	Stable sleep and wake timing may reduce behavioral fragmentation	Tier 2; candidate	General sleep care is reasonable; timing-specific bone–muscle benefit unvalidated	Diagnose sleep disorders; review sedating drugs and fall risk
Protein distribution	Adequate protein distributed across meals and around training may support anabolic responses	Tier 2; candidate	Adequate intake is standard care; optimal timing unvalidated	Kidney disease, low appetite, dysphagia and total energy adequacy
Time-restricted eating	Active-phase eating may alter glucose, lipid and microbial patterns	Tier 2; investigational	Not recommended on timing grounds outside supervised research	Frailty, low body mass index, malnutrition, diabetes medication, kidney disease, and inadequate protein, calcium or micronutrients
Exercise timing	Timing matched to chronotype and goals may modify metabolic and loading responses	Tier 2–3; candidate	Exercise is standard care; optimal time unvalidated	Falls, cardiovascular risk, sleep disruption, fatigue and adherence
Medication timing	Clinician-directed schedules may test timing-dependent exposure	Tier 3–4; speculative	Do not change prescribed schedules outside clinician-led care or research	Polypharmacy, interactions, kidney function and indication-specific safety

## Data Availability

No new data were created or analyzed in this study. Data sharing is not applicable to this article.

## Abbreviations

The following abbreviations are used in this manuscript:

BAIBA	beta-aminoisobutyric acid
BCAA	branched-chain amino acid
BMAL1	brain and muscle ARNT-like 1
BMI	body mass index
CGM	continuous glucose monitoring
CRP	C-reactive protein
CTX	C-terminal telopeptide of type I collagen
DXA	dual-energy X-ray absorptiometry
EV	extracellular vesicle
FGF23	fibroblast growth factor 23
FNDC5	fibronectin type III domain-containing protein 5
GLUT4	glucose transporter type 4
HIF1α	hypoxia-inducible factor 1 alpha
HRV	heart-rate variability
IGF-1	insulin-like growth factor 1
IL	interleukin
mTORC1	mechanistic target of rapamycin complex 1
NAD^+^	nicotinamide adenine dinucleotide
OPG	osteoprotegerin
P1NP	procollagen type I N-terminal propeptide
PGC1α4	peroxisome proliferator-activated receptor gamma coactivator 1 alpha 4
PTH	parathyroid hormone
RANKL	receptor activator of nuclear factor-kappa B ligand
SCFA	short-chain fatty acid
SCN	suprachiasmatic nucleus
TGF-β	transforming growth factor beta
TNF-α	tumor necrosis factor alpha
TRE	time-restricted eating

## Appendix A. Search Strategies and Reporting Items

Table A1 reports the Boolean logic used in the four core bibliographic databases and explains how supplementary platforms were used. Syntax was adapted to the fields and operators supported by each interface. Searches covered database inception to 28 June 2026; no study-design filter was applied. Narrower combinations of the same concepts were used to resolve section-specific mechanistic questions. Because this narrative workflow did not retain a stable deduplicated record log, source-level yields and a retrospective flow count are not reported. Table A2 distinguishes the procedures used from those not performed, and Table A3 lists reporting items for future chronometabolomic studies.

**Table A1:** Source-specific search strategies and source roles for the structured narrative review.

Source	Search Syntax or Approach	Coverage and Role
PubMed/MEDLINE	(osteosarcopenia OR sarcopenia OR osteoporosis OR osteopenia OR “bone muscle crosstalk”) AND (circadian OR chronotype OR “biological clock” OR sleep OR “rest activity rhythm” OR “meal timing” OR “exercise timing”) AND (metabolomics OR lipidomics OR metabolite OR glucose OR “amino acid” OR lipid OR mitochondrial OR redox OR melatonin OR microbiota)	Inception–28 June 2026; titles, abstracts and indexed terms; core source
Web of Science Core Collection	TS=((osteosarcopenia OR sarcopenia OR osteoporosis OR osteopenia OR “bone-muscle crosstalk”) AND (circadian OR chronotype OR “clock gene” OR sleep OR “rest-activity rhythm” OR chrononutrition OR chronotherapy) AND (metabolomics OR lipidomics OR “rhythmic metabolite*” OR mitochondria OR redox OR microbiota))	Inception–28 June 2026; SCI-Expanded included; core source
Embase	(’osteosarcopenia’/exp OR sarcopenia OR osteoporosis OR osteopenia OR ’bone muscle crosstalk’) AND (circadian OR chronotype OR ’biological clock’ OR sleep OR ’rest activity rhythm’ OR ’meal timing’ OR ’exercise timing’) AND (metabolomics OR lipidomics OR metabolite OR glucose OR ’amino acid’ OR lipid OR mitochondrial OR redox OR melatonin OR microbiota)	Inception–28 June 2026; mapped terms and free text; core source
Scopus	TITLE-ABS-KEY((osteosarcopenia OR sarcopenia OR osteoporosis OR osteopenia OR “bone-muscle crosstalk”) AND (circadian OR chronotype OR “clock gene” OR sleep OR “rest-activity rhythm” OR “meal timing” OR “exercise timing”) AND (metabolomics OR lipidomics OR metabolite* OR glucose OR “amino acid*” OR lipid* OR mitochondri* OR redox OR melatonin OR microbiota))	Inception–28 June 2026; title, abstract and keywords; core source
ScienceDirect	Targeted combinations of the same phenotype, timing, pathway and intervention terms	Supplementary full-text discovery and verification; not treated as an independent exhaustive record set
Google Scholar	Targeted phrase searches and cited-by searching for eligible or foundational articles	Supplementary citation discovery; relevance ranking and interface instability precluded reproducible exhaustive yields
Reference chaining	Backward reference review and forward citation searching from directly relevant reports	Located primary studies and recent related reports

**Table A2:** Review conduct and methodological boundaries.

Item	Approach Used	Boundary
Review design	Structured narrative synthesis informed by SANRA domains	Not a systematic review or meta-analysis; no prospective registration
Eligibility	Peer-reviewed human or experimental evidence addressing at least two prespecified domains; reviews used for context and citation discovery	Broad eligibility reflects an intentionally integrative question
Screening	Title/abstract relevance assessment followed by full-text assessment; purposive selection based on directness, design, temporal resolution and translational relevance	No retained auditable deduplicated record log or PRISMA flow count
Evidence interpretation	Four-tier directness framework with explicit candidate, unvalidated or speculative status	Not a formal certainty-of-evidence system
Quality assessment	Study design, population, sampling and directness considered when calibrating claims	No duplicate independent screening or formal risk-of-bias tool
Synthesis	Qualitative comparison of clinical, chronobiological, metabolomic and mechanistic evidence	Heterogeneity precluded quantitative pooling

**Table A3:** Recommended reporting items for future chronometabolomic osteosarcopenia studies.

Item	Minimum Information	Reason
Musculoskeletal phenotype	Named guideline and version, osteoporosis/osteopenia or fragility-fracture criteria, DXA site and threshold, sarcopenia algorithm, lean-mass method, strength and performance cutoffs, and fracture/fall history	Separates isolated and combined phenotypes and enables comparison across definitions
Sampling time	Clock time, fasting duration, prior meal, posture, medication timing, recent activity	Prevents time-of-day misinterpretation
Circadian context	Chronotype, sleep midpoint, sleep duration, sleep regularity, light exposure, rest–activity rhythm	Links clock time with biological time
Nutrition/activity context	Eating window, protein distribution, exercise mode, exercise timing, sedentary time, step counts	Connects metabolites with major zeitgebers
Analytical strategy	Predefined rhythm variables, covariates, missing-data handling, multiple-testing control, pathway analysis	Supports reproducible rhythm interpretation
